# A Modified Method for Isolation of Rhein from Senna

**DOI:** 10.4103/0250-474X.54275

**Published:** 2009

**Authors:** Namita Mehta, K. S. Laddha

**Affiliations:** Medicinal Natural Products Research Laboratory, Pharmaceutical Sciences Division, Institute of Chemical Technology, Nathalal Parikh Marg, Matunga, Mumbai-400 019, India

**Keywords:** Sennosides, rhein, aloe-emodin, *Cassia angustifolia*

## Abstract

A simple and efficient method for the isolation of rhein from *Cassia angustifolia* (senna) leaves is described in which the hydrolysis of the sennosides and extraction of the hydrolysis products (free anthraquinones) is carried out in one step. Further isolation of rhein is achieved from the anthraquinone mixture. This method reduces the number of steps required for isolation of rhein as compared to conventional methods.

Rhein (1,8-dihydroxyanthraquinone-3-carboxylic acid) is a compound found in the free state and as a glucoside in *Rheum* species, senna leaves; and also in several species of *Cassia*[1]. Rhein is currently a subject of interest because of its antiviral, antitumor and antioxidant properties. It also serves as a starting compound for the synthesis of diacerein (1,8-diacyl derivative, fig. 1), which has antiinflammatory effects and is useful in the treatment of osteoarthritis. Therefore, there is a need for a simple and efficient extraction method to obtain rhein from indigenous plant sources[2]. Currently the preferred method for synthesis of diacerein is by oxidative hydrolysis of aloin (a C-glycoside present in *Aloe* species) to obtain aloe-emodin, acetylation of aloe-emodin and subsequent chromic oxidation of the acetylated product to obtain diacerein[2].

**Fig. 1:**
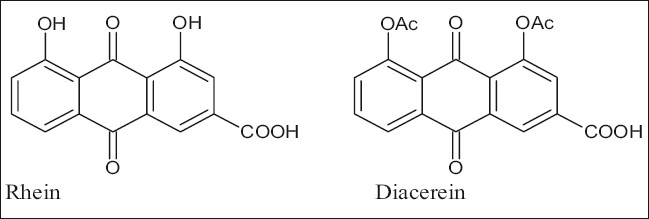
Rhein and its 1,8-diacyl derivative (diacerein)

Senna (*Cassia angustifolia*) is a small shrub that is widely cultivated in southern India, mainly in Tamil Nadu. The leaves and pods of this plant contain not less than 2.5% of anthraquinone glycosides mainly sennosides A and B (fig. 2), that are dianthrone glucosides derived from rhein and aloe-emodin. This makes senna leaf an important source of rhein[3]. The following paper describes a simple method for the isolation of rhein from senna leaf.

**Fig. 2:**
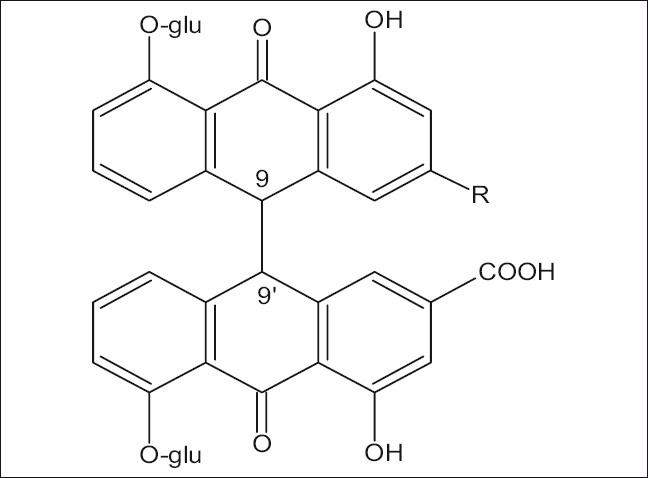
Anthraquinone glycosides (sennosides) present in senna leaves. For sennoside A, R=COOH (threo form) and for sennoside B, R=COOH (erythro form). For sennoside C, R=CH_2_OH (threo form), and for sennoside D, R=CH_2_OH (erythro form)

The leaves of *C. angustifolia* were obtained from the local market, Mumbai. Sodium hydrogen carbonate and hydrochloric acid were of analytical grade and were purchased from S. D. Fine Chemical Limited, Mumbai. The leaves were powdered and the powdered leaves used for extraction purpose.

To 25 g powdered senna leaves a mixture comprising of 75% water in alcohol was added. The mixture was warmed slightly and to it added 5 ml of hydrochloric acid. One hundred millilitres of toluene was then added to form a biphasic mixture and refluxed for 6 h. At the end of the 6 h the mixture was cooled slightly and filtered to remove any crude drug and the aqueous and organic layers separated from each other. The crude drug and aqueous layers were washed with toluene in order to recover any free anthraquinones and the toluene layers combined together. The toluene layer was partitioned with 10% aqueous sodium hydrogen carbonate solution until the aqueous layer ceased to show the characteristic pink colour. The aqueous layer was acidified with hydrochloric acid, and the precipitate taken up in ethyl acetate. The ethyl acetate layer was evaporated and the product recrystallized from glacial acetic acid. A dark yellow compound was obtained that was subjected to chemical tests and spectral studies in order to establish its identity. An alcoholic solution of the isolated compound was treated with Borntrager's reagent (5% alcoholic potassium hydroxide); a pink colour was obtained indicating the presence of anthraquinones[4].

Thin-layer chromatography was performed on pre-coated silica gel G60 F_254_ plate (E. Merck) using ethyl acetate:methanol:water as the mobile phase, a single band was seen at R_*f*_ 0.3, which gave a pink colour with Borntrager's reagent. Infra red (IR) spectrum of the isolated compound was recorded on a Perkin-Elmer FTIR spectrometer. The IR spectra showed a broad peak at 3063 cm^−1^ (hydroxyl), 1629 cm^−1^ (chelated carbonyl) and 1696 cm^−1^ (carboxyl). Mass spectrum, was recorded on a Micromass, Q-TOF MS ES+. Molecular ion peak at 285 *m/e* gave the molecular weight of the compound. The UV/Vis spectrum was recorded on a Jasco V-530 UV/Vis Spectrophotometer. The UV/Vis maxima in methanol (nm) were found at 228, 258 (Ar-C=O) and 432 (quinonoid group)[5]. Based on chemical tests and spectral studies, the isolated compound was identified as rhein.

The method described above employing a biphasic system to hydrolyze and extract anthraquinones in a single step was found to be efficient on a laboratory scale for the isolation of rhein. The rhein so obtained can be used for the synthesis of diacerein and other derivatives of rhein. Further optimization of this method is necessary to make it suitable for large-scale extraction.
